# Conservative Operation of the Ovaries

**Published:** 1918-01

**Authors:** H. Stewart MacLean

**Affiliations:** Richmond, Va.


### CONSERVATIVE OPERATION OF THE OVARIES.

**H. Stewart MacLean, M.D., Richmond, Va.**

It is unfortunate that surgery has been divided into conservative and radical. There should be, indeed there is, but one kind of real surgery, and that will always be conservative. We may have conservative and radical operation, but the most radical operation when justified by the exigencies of the case becomes conservative surgery. Proper surgery always has for its objectives conservation of life, health and function. In the field of pelvic surgery this subject has received especial emphasis. For a time radicalism predominated, largely because surgeons were dealing with an indefinite symptomatology and hoped more or less blindly to evolve a startling panacea for a trying class of cases. Psychic cases of indefinite symptomatology were referred by troubled physicians to the surgeon as a last resort, and the surgeon seized upon a few vague evidences of pelvic disorder from out the mass of jumbled symptomatology and operated, too often to his own discomfort and discredit. At one time double oophorectomy was commonly performed for vague and uncertain indications and was justified on more vague and more uncertain pathology. Because an ovary was small and irregular, or it may be, somewhat enlarged, or contained one or more systs, it was gravely indicted, condemned and removed. It really seemed at time as though some surgeons had little conception of what constituted a normal ovary, much less what abnormality constituted sufficient reason for removal. Our old acquaintances in the surgical feld, cirrhotic and cystic ovary, have been bereft of their pathological and surgical importance. An indefinite train of nervous and mental symptoms were blamed on these ovaries for want of a more convenient "goat," but such ovaries and such symptoms have been effectually divorced in the court of sad and bitter experience, although even now surgeons are still performing radical and unjustifiable operations on the ovaries.

In order to approach the subject satisfactorily, we must first review our knowledge of the function of the ovary. As a functionating organ it has been considered simply as concerned in ovulation and, through the elaboration of a harmone, menstruation. We cannot explain the comparatively abrupt cessation of menstruation at the menopause on any known histological or physiological basis. Norris (Am. J. obs., 1910, LXI, 203) has attempted to establish cessation of menstruation on the ground of the formation in the ovarian cortex of scar tissue, and Culberston in a recent article quotes Norris as elaborating this theory into an explanation as to why the multipara arrives at the menopause later than the nuuipara. The periods of gestation and lactation produce a corresponding suspension of ovulation and hence fewer corpor albicantes are formed. But when we come to apply this theory in some of our surgical cases, it will not stand test; for instance, we may have occasion to remove one and a half ovaries from a women between 20 and 25 years of age, yet menstruation will continue and will be expected to run to the normal time for the menopause.

Even if these were the only functions of the ovary, we would have every incentive to preserve ovarian tissue. The right of conception and motherhood must not lightly be denied to any woman. When we deliberately remove ovaries for a lesion which manifestly has not entirely destroyed their functioning value, and for the cure of symptoms concerning which there is considerable doubt that they arise from that lesion, we fail to exercise that reasonable judgment which the patient has a right to expect and demand.

A conservative operation may always be supplemented by a second; a radical cannot. The fact that menstruation will never return exerts a profound mental influence at times despite the patient's apparent willingness before the operation to "have everything taken out." The ill-advised wishes of the patient must be resisted as well as the radical inclination of the sugeon "to take a chance."

There are excellent reasons for believing that the ovaries are intimately concerned in internal secretion, and the evidence is almost all in favor of such relationship. The experiments of Loewy and Ritcher (Arch. of Physiol. 1889, p. 174) and others show conclusively that the ovaries form a specific substance which inceases oxidation of the tissues. This agent enhances metabolism, increases the hemoglobin content of the blood and produces an increase in respiratory exchange. Graves (Am. Jour. Obs., 1913) states, "after sexual maturity the ovary exercises a trophic influence over the other internal and external genital organs." Novak (Journal of the A. M. A., October 28, 1916) assigns to the corpus luteum the cause or control of menstruation and emphasizes that it works in association with other ductless glands of the body. Heimann (Int. Obstr. Surg., December, 1913) ascribes to internal ovarian secretion the property of reducing the number of lymphocytes, showing a variation of from 18 to 22 per cent. in their number at menstruation and between periods. To say the least, the evidence is too conflicting to justify a positive opinion that the ovary performs no function after the menopause. It is well known that those cases of double oophorectomy followed by marked nervous and mental symptoms have a higher percentage of persistence of serious nervousness and mental disorders than would be found in a similar number of non-surgical climacterics.

Gordon (Journal of the A. M. A., October 17, 1914) in his study of 112 cases brings out some very interesting figures for our consideration. In 34 of his cases double oophorectomy was performed for vague disturbances in the obdomen, together with a train of indefinite nervous symptoms. None of these patients were permanently benefited. Nine showed an immediate improvement in the preexisting functional disorders, but shortly thereafter the former conditions returned. "In all the cases the former nervous and psychic phenomena have been aggravated in weeks and months following the operation." In 75 out of the 112 cases, complete hystero-oophorectomy was performed. "The removal of the diseased organs removed the localized discomfort but in the months following the operation, symptoms of a more distressing nature began to develop. In the large majority of my patients the former nervousness not only remained but became accentuated." Among his conclusions I would quote the following: "As in the removed organs healthy portions of tissue were invariably found, it is to be supposed that the removal of the latter is in some relation to the morbid phenomena observed after the operations. The logical conclusion seems to be that one must be very cautious in advising operation on the generative organs and the tendency should be to oppose as much as possible the removal of any amount of normal tissue found in the uterus or ovaries. No operation should be advised on healthy organs if a woman complains of vague nervous symptoms."

This article is particularly valuable as it gives us a view of some of the end-results and what the family physician has to contend with in the after-care of those cases which have had double oophorectomy and made a satisfactory surgical recovery. It has long been the contention that while it was right and proper to practice conservation when dealing with diseased ovaries alone there is no use in considering the ovaries or in making any special effort to retain them or any portion of them when the uterus is removed, as the latter operation effectually terminates menstruation and renders the ovaries useless and negligible. Here is where we believe surgical treatment has been bad and has been contrary to what we can assume to be the character of the ultimate ovarian function. If we cannot be absolutely certain that the ovaries have no function beyond ovulation and menstruation we must not sacrifice those organs as a matter of expediency, or because their removal is a feature of a well-developed technic. I have reviewed the last 62 hysterectomies for disease of the uterus in women who had not yet reached the climacteric. None of these cases had both ovaries removed and less than half of them had any ovarian tissue removed. Forty-nine of the cases have been followed and in only one case is it reported that the patient suffered any excessive or prolonged nervous and mental symptoms. In this one case, the family physician reports that about seven months after the operation she became very nervous and depressed, developing a mild melancholia which persisted for about ten months, when she apparently recovered.

We have no reason to believe that the function of the ovaries in contributing to internal secretion does not continue after the menopause; in fact, it would be unreasonable to suppose that such is not the case. In a period of over eighteen years I have made it a rule never to remove both ovaries completely from any patient without at least making the effort to retain a portion of comparatively normal ovarian tissue from one of the diseased organs. Exception, of course, is made in cases of malignant tumors, but otherwise, irrespective of the age of the patient, some ovarian tissue has always been left. In hysterectomy for uterine diseases both the ovaries are invariably left, irrespective of the patient's age, unless they are so badly diseased as to be a menace to the patient's health. The mere fact that an ovary contains one or more cysts is no reason for the removal of that organ.

Considering the large number of cases in which we find small multiple cysts in one or both ovaries and the comparatively small number that attain great size, we are forced to the conclusion that most of these cysts rupture spontaneously or may possibly be absorbed, the determining factors as to the size of an ovarian cyst probably being the thickness of the limiting membrane and the degree of vascularity or nourishment of the organ. It is doubtful if these small cystic ovaries are harmful or even painful to the patient. Usually it is only when they attain such size as to be mechanically troublesome that it is necessary to resect the cyst or remove the ovary. Exception would be made, of course, in the case of such ovaries, which by reason of inflammatory conditions, adhesions, or otherwise are sensitive and the source of pain and distress. It is very seldom that we will have to deal with a case of cystic degeneration of the ovary so extensive that a portion of the ovarian tissue connot be found somewhere near the pedicle sufficiently normal and of sufficient size to warrant its retention. Not infrequently ovarian tissue which might be saved is sacrificed rather than lose the opportunity of removing a big cyst intact for the purpose of demonstration. It should be borne in mind that such portions of ovarian tissue as may be thus saved will probably be deficient in blood supply on account of the ligatures which it has been necessary to place about the pedicle. It is, however, an easy matter to cover them with amentum, or better still, cover them with a piece of peritoneum from the broad ligament, thus assuring a new blood supply in case the old should be obliterated.

Conclusions: The ovary is probably vitally concerned in the scheme of internal secretion and while such function may gradually decrease after 54 years of age as do the activities of many of the other organs of internal secretion, yet we have no reason for believing that such function ceases abruptly at the menopause.

The fact that nervous and mental symptoms following artificial menopause are apt to be more violent and more lasting that those following the natural monopause is additional evidence that the ovary has been concerned in something else besides ovulation and menstruation.

Irrespective of the age of the patient some ovarian tissue should always be left.—American Journal of Surgery.

